# Supplementary material for: Predicting the hosts of prokaryotic viruses using GCN-based semi-supervised learning
Source: BMC Biol. 2021 Nov 24;19:250. doi: 10.1186/s12915-021-01180-4 (PMC8611875; doi:10.1186/s12915-021-01180-4)
Supplement: Supplementary file 1 — Additional file 1 Fig S1 –[Prediction performance of BLASTN at the order, family, and genus level. X-axis: taxonomic level. Y-axis: Accuracy.] Fig S2 – [Associations between accuarcy and similarity at genus level. X-axis: Mash distance. Left Y-axis: Accuracy. Right Y-axis: number of testing virus.] Fig S3 – [Prediction performance of HostG at the order, family, and genus level. X-axis: Prediction rate. Y-axis: Accuracy.] Tables S1-S2. Table S1 –[Parameters used for training CNN and GCN.] Table S2 – [Elapsed time for each tool. All the methods were run on Intel® Xeon® Gold 6258R CPU with 8 cores.] [file 12915_2021_1180_MOESM1_ESM.pdf]

SUPPLEMENTARY FILE 1

Detecting the hosts of prokaryotic viruses using GCN-based semi-supervised learning

Jiayu Shang and Yanni Sun*

*Correspondence:
yannisun@cityu.edu.hk
Electrical Engineering, City University of Hong Kong, Hong Kong, China SAR
Full list of author information is available at the end of the article

1 Parameters used in the model

**Convolutional nerual network**	
Filter size	[3, 7, 11, 15]
no. of convolutional layer	1
no. of dense layer (hidden unit)	2 (512, 256)
learning rate	0.01
**Graph convolutional nerual network**	
no. of convolutional layer	2
no. of dense layer (hidden unit)	1 (32)
learning rate	0.01

Table S1: Parameters used for training CNN and GCN

Table S1 shows the default parameters used for training CNN and GCN. Because the number of hidden units in the first dense layer of CNN is 512, the encoded node feature will be 512-dimensional vectors.

Detail description of skip-gram model For each position i on the segment, the 3-mer at position i will be used as input and its' neighboring 3-mer in range $[i - j, i + j]$ will be utilized as output. j is a hyperparameter that can be adjusted for the skip-gram model. Since we employ 100 hidden units in the embedding layer to encode each 3-mer, 3-mers on the segments will be converted into 100-dimension vectors. Thus, each 2kbp segment will be converted into a matrix $X \in \mathbb{R}^{1,998\times100}$.

2 Elapsed time

Program	HostG	PHP	WIsH	VHM-net	BLASTN	HoPhage	VPF-Class	RaFaH	vHULK
Elapsed time (min/100 phages)	11	3	2	8	7	8	10	10	17

Table S2: Elapsed time for each tool. All the methods were run on Intel® Xeon® Gold 6258R CPU with 8 cores.

HostG has longer running time than other tools as shown in Table S2. The bottleneck of HostG is the calculation of the alignment similarities. We will explore whether the alignment step can be replaced by a more efficient method to save computational resources.

3 10-fold cross validation on VHM dataset

In this experiment, we applied 10-fold cross-validation to split the training and validation sets on the VHM dataset. We randomly separate the VHM dataset into

10 subsets. Then we trained HostG on 9 subsets and validate the results on 1 subset iteratively. Finally, we keep the model with the highest accuracy.

4 Prediction results of BLASTN

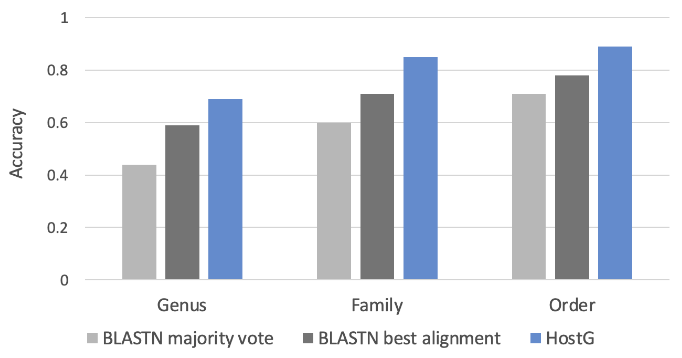

Figure S1: Prediction performance of BLASTN at the order, family, and genus level. X-axis: taxonomic level. Y-axis: Accuracy.

As shown in Fig. S1, The majority vote strategy assigns the most common alignment host to the virus. The best alignment strategy predicts the host with the best alignment. The results show that predicting with the best alignment is better than the majority vote method. This might because the numbers of hosts in each taxonomic group are unbalance and the majority vote tend to predict a taxonomic group with more genomes. Thus, the performance of this method highly depends on the distribution of the reference database.

5 Prediction results of RaFAH and vHULK using their pre-trained models

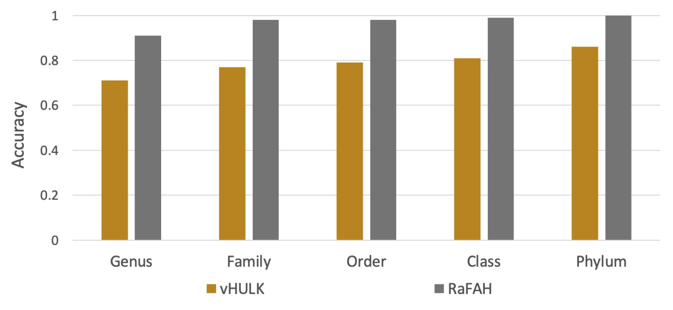

Figure S2: Prediction performance of RaFAH and vHULK with pre-trained parameters. X-axis: taxonomic level. Y-axis: Accuracy.

Fig. S2 shows the results of RaFAH and vHULK using their pre-trained models. The difference between Fig. S2 and Fig. 6 in the main article is likely caused by the overlap between the TEST dataset and the data used for training the latest RaFAH and vHULK models. For example, according to the description of RaFAH, the model was built using genomes before Oct. 2019, indicating a large overlap with the TEST dataset.

6 Impact of the sequence similarity between the training and testing samples on the prediction performance

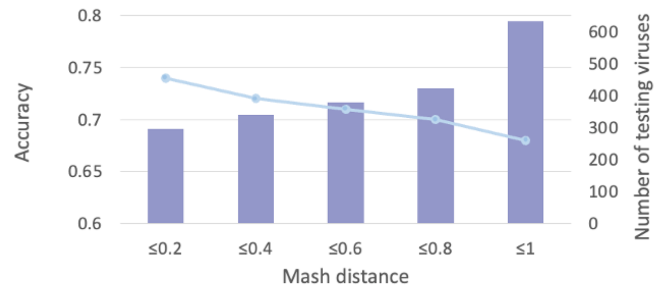

Figure S3: Associations between accuracy and similarity at the genus level. X-axis: Mash distance. Left Y-axis: Accuracy. Right Y-axis: number of testing viruses.

First, we used Mash [1] to calculate the distance between sequences in training and testing sets. For each virus in the testing set, we recorded its smallest distance to the training phages. The results show that the smallest distance of 27% phages is 1, which means they are significantly different from all training phages. The average distance is 0.54, and no virus got a 0 distance. Then, we sorted the host prediction by the Mash distance and reported the results in Fig. S3. X-axis stands for the upper-bound cutoff of the distance between genomes in the training set and testing set. For example, when the X-axis value is 0.2, this means that all the genomes in the test and train have mash distance ≤ 0.2. With the increase of the distance, more testing genomes with lower similarities are included, and the accuracy (Y-axis) decreases as expected. We also showed the number of testing viruses satisfying the distance cutoff.

7 Improvement of GCN with ECE

We first sorted the prediction according to the SoftMax value and then showed the results in Fig. S4. As expected, the accuracy tends to decrease with the increase of the prediction rate. In addition, HostG achieves 100% accuracy at the order, family, and genus level when the SoftMax thresholds are 0.88, 0.89, and 0.94, respectively.

We also record the trend of F1-score in Fig. S5. The prediction rate represents the number of viruses which have predictions and it is the same as the definition of recall in other methods. As shown in Fig. S5, with the increases of the prediction rate (recall), the F1-score increases, and HostG can achieve higher F1-score than most of the existing tools under the same prediction rate.

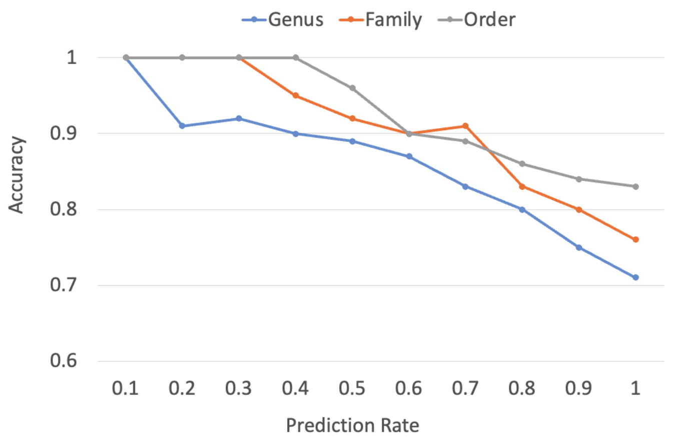

Figure S4: Prediction performance of HostG at the order, family, and genus level. X-axis: Prediction rate. Y-axis: Accuracy.

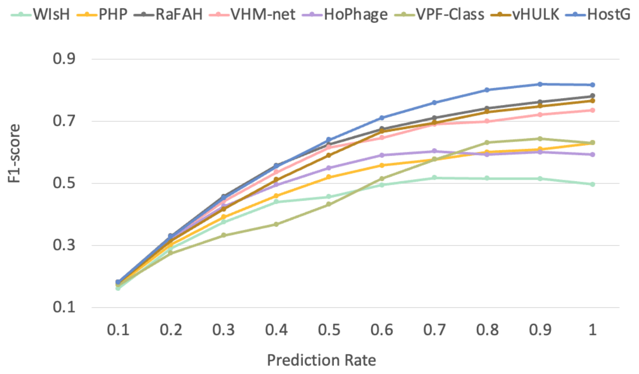

Figure S5: Comparison of the F1-score and prediction rate (recall) for the learning-based tools at the rank of genus. Each data point on a line corresponds to a different confidence threshold. X-axis: prediction rate (recall). Y-axis: F1-score.

8 The commands and parameters of running other tools

All the parameters used in the command are default parameters suggested by their guidelines.

8.1 HoPhage

```
python predict.py -q test_phages.fa -c phage_cds.fna -o output_example
    -w 0.5 -g candidate_host_genera.csv --all
```

8.2 VPF-Class

```
stack exec -- vpf-class --data-index ../data/index.yaml -i ../data/
    test_phages.fa -o test-classified
```

8.3 VHM-net

```
python VirHostMatcher-Net.py -q test_phage/ -o output -i tmp -n 1 -t 8
```

8.4 PHP

```
python countKmer.py -f ./HostGenome -d ./Output -n HostKmer -c -1
python PHP.py -v ./test_phage/  -o ./Output  -d ./Output  -n HostKmer
```

8.5 WIsH

```
./WIsH -c build -g ./HostGenome/ -m modelDir
./WIsH -c predict -g ./test_phage/ -m modelDir -r outputResultDir -b 1
```

8.6 RaFAH

```
perl RaFAH.pl --train --genomes_dir train/ --extension .fasta --
    true_host Genomes_Hosts.tsv --file_prefix Custom_Model_1 --threads
     32
perl RaFAH.pl --predict --genomes_dir test/ --extension .fasta --
    file_prefix test
```

8.7 vHULK

```
python vHULK.py -i test_input -o test_output -t 4
```

Author details
Electrical Engineering, City University of Hong Kong, Hong Kong, China SAR.

References

1. Ondov, B.D., Treangen, T.J., Melsted, P., Mallonee, A.B., Bergman, N.H., Koren, S., Phillippy, A.M.: Mash: fast genome and metagenome distance estimation using minhash. Genome biology **17**(1), 1–14 (2016)
